# An Air Particulate Pollutant Induces Neuroinflammation and Neurodegeneration in Human Brain Models

**DOI:** 10.1002/advs.202101251

**Published:** 2021-09-24

**Authors:** You Jung Kang, Hsih‐Yin Tan, Charles Y. Lee, Hansang Cho

**Affiliations:** ^1^ Department Mechanical Engineering and Engineering Science Department of Biological Sciences Center for Biomedical Engineering and Science University of North Carolina at Charlotte Charlotte NC 28223 USA; ^2^ Institute of Quantum Biophysics Department of Biophysics Sungkyunkwan University Suwon‐si Gyeonggi‐do 16419 ROK; ^3^ Institute for Health Innovation & Technology National University of Singapore Singapore 117599 Singapore; ^4^ Department of Intelligent Precision Healthcare Convergence Sungkyunkwan University Suwon‐si Gyeonggi‐do 16419 ROK

**Keywords:** astrocytes, human brain model, microglia, neuroinflammation, particulate matter

## Abstract

Fine particulate matter (PM2.5), a major component among air pollutants, highlights as a global health concern. Several epidemiological studies show the correlation between chronical PM2.5 exposure and incidents of neurological disorders including Alzheimer's disease. However, the mechanisms have not been well understood, partly due to the lack of model systems that reflect the physiologically relevant innate immunity in human brains. Here, PM2.5‐polluted human brain models (PMBs) are created in a 3D microfluidic platform reconstituting key aspects of human brain immunity under the PM2.5 exposure. PM2.5 penetration across a blood–brain barrier (BBB) model and accumulation in the brain tissue side of the model are first validated. Second, the PMB model shows that the BBB‐penetrating PM2.5 initiates astrogliosis, resulting in slight neuronal loss and microglial infiltration. Third, it is demonstrated that the infiltrating microglia obtain M1 phenotype induced by interleukin‐1*β* and interferon‐*γ* from neurons and reactive astrocytes under the PM2.5 exposure. Finally, it is observed that additional proinflammatory mediators and nitric oxide released from the M1 microglia exacerbate neuronal damages, such as synaptic impairment, phosphoric tau accumulation, and neuronal death. This study suggests that PM2.5 can be a potential environmental risk factor for dementia mediated by the detrimental neuroinflammation.

## Graphical abstract

Fine particulate matter (PM2.5) is a major component among air pollutants highlighted as a global health concern. Here, microfluidic PM2.5‐polluted human brain models are created for the study of human innate immunity responding to the PM2.5 exposure. This study suggests that PM2.5 can induce the detrimental microglial proinflammation and therefore be a potential risk factor for neurodegeneration.

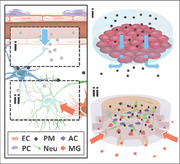

## 1 Introduction

Particulate matter (PM) is a major component of urban air pollutions and has been highlighted in the Global Burden of Disease (GBD) 2015 as a major global health concern.^[^ ^1^ ^]^ Fine particulate matter (PM2.5, aerodynamic diameters less than 2.5 µm) resulted in more than 4.2 million deaths accounting for 7.6% cause of deaths worldwide in 2016.^[^ ^2^ ^]^ Particularly, PM2.5 obtained from diesel exhaust can be more toxic as it can absorb chemicals including polycyclic aromatic hydrocarbons (PAHs) and nitro‐substituted PAHs (nitro‐PAHs), which enhance inflammatory responses.^[^ ^3^ ^]^ While PM2.5 has been strongly correlated with increased risk for pulmonary and cardiovascular diseases,^[^ ^4^ ^]^ it is now acknowledged as an enhanced risk factor for neuroinflammation that may damage the central nervous system (CNS).^[^ ^5^ ^]^ According to significant number of epidemiological studies, individuals residing in the areas highly polluted with PM2.5 tended to have an increased neuroinflammation in the brain tissue, warning the strong association between air pollution and brain damage.^[^ ^5h^ ^]^ Recent studies revealed that inhaled PM2.5 can affect brain health by transducing proinflammatory signals originating from peripheral tissues of lung, liver, and cardiovascular system.^[^ ^4^ ^]^ In addition, the significant accumulation of PM2.5 in the brain areas has been observed, which directly interact with brain immune surveillance and may increase neuroinflammation.^[^ ^5^ ^]^ A number of studies reported that the neuroinflammation induced by PM2.5 can enhance risk of other CNS disorders such as Alzheimer's disease (AD),^[^ ^5b,h^ ^]^ Parkinson's disease (PD),^[^ ^5c,d^ ^]^ brain stroke,^[^ ^5e,f^ ^]^ and dementia.^[^ ^5g^ ^]^ In this regard, it is important to understand the pathophysiology of brain damage caused by PM2.5 and their contribution to other neurological disorders.

Currently, various in vivo animal models exposed to PM2.5 through respiratory system have been extensively used to elucidate the neuroinflammation driven by the inhaled PM2.5. Consistent with human studies, these animal model studies confirmed that the inhalation of PM2.5 elevated sources of oxidative stress (e.g., hydrogen peroxide (H_2_O_2_) and nitric oxide (NO)), and proinflammation (e.g., interleukin‐1*β* (IL‐1*β*), interleukin‐6 (IL‐6), tumor necrosis factor‐*α* (TNF‐*α*), interferon‐*γ* (IFN‐*γ*), and complements), in the brain that may lead to neurodegeneration.^[^ ^6^ ^]^ In addition, hyperactivated immune cells, including monocyte,^[^ ^7^ ^]^ microglia,^[^ ^6a–e^ ^]^ T cells,^[^ ^8^ ^]^ and astrocytes,^[^ ^6c,f^ ^]^ were observed in PM2.5‐treated brain tissues. Among the immune cells, astrocytes, the most abundant glial cells providing immune surveillance, acquired an increased reactivity that further elevate oxidative stress and inflammation in the brain.^[^ ^6c,f^ ^]^ Other independent studies revealed that microglia, resident myeloid cells of CNS, were recruited to the olfactory bulb or other brain regions and promoted significant oxidative stress and proinflammation.^[^ ^6a–e^ ^]^ However, the correlation between astrogliosis and microgliosis under PM2.5 environments contributing to adverse neuroinflammation remains unclear partly due to limited model systems that accurately reflect the innate immunity from the molecular to the cellular level.^[^ ^9^ ^]^ Moreover, these animal models have different genetic backgrounds from humans. Therefore, humanized model systems are highly in demand to resolve the underlying mechanisms of PM‐driven neuroinflammation.

Here, we aim to elucidate the cross talk of astrocytes and microglia resulting in neurodegeneration in the air‐pollutant‐exposed brains by using a 3D cultured model in the microfluidic system that faithfully reproduces the interactive environments between these glial cells and neurons. We particularly examined the effects of PM2.5 from diesel exhaust on our human brain models through this study. We first validated the penetration of PM2.5 through in vitro blood–brain barrier (BBB) models and determined the concentration of PM2.5 accumulating in the tissue areas. We then built two types of microfluidic PM2.5‐polluted brain models, comprised of two compartments: a brain tissue compartment where human neuron and astrocytes were exposed to PM2.5 and a compartment where resting microglia reside. We found the increased reactivity of astrocytes under PM2.5 conditions producing proinflammatory chemokines and cytokines that recruit microglia and further induce polarization of M1 microglia serving neurodegenerative roles. Finally, we validate microglial activities exacerbating neurodegeneration under PM2.5 conditions. This study will offer transformative information that may contribute to the development of promising preventive or curable therapeutics for PM2.5‐driven neurological disorders.

## 2 Results

### 2.1 Penetration of PM2.5 across In Vitro BBB Model

**Figure 1:**
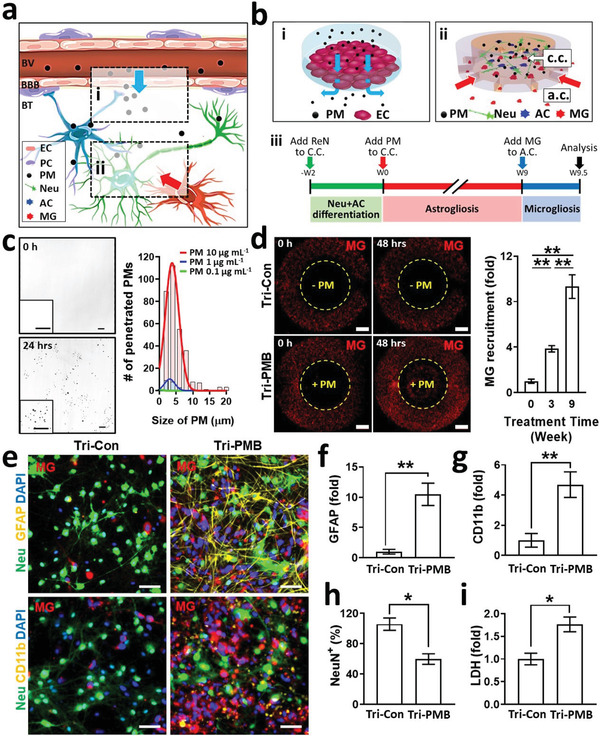
Generation and characterization of human brain models exposed to PM2.5. a) Schematic representation of BBB‐penetrating PM2.5 causing neurodegeneration in brains: i) PM2.5 penetration across BBB (blue arrow) and ii) PM2.5‐driven neuroinflammation (red arrow). BV represents a blood vessel; BBB, a blood–brain barrier; BT, a brain tissue; EC, an endothelial cell; PC, a pericyte; PM, fine particulate matter; Neu, a neuron; AC, an astrocyte; MG, microglia. b) Schematic illustration of human brain models to investigate: i) PM2.5 penetration across BBB and translocation to brain tissue and ii) PM2.5‐driven neuroinflammation by astrocytes and microglia; c.c. represents a central chamber; a.c., an annular chamber; iii) timeline of the Tri‐PMB preparation. We replaced the one half of control or PM2.5 diluent every 3.5 days. c) Validation of BBB penetration. Phase contrast images show the amount of PM2.5 detected from the brain side of the BBB model prepared in the Trans‐well system exposed to PM2.5 at 10 µg mL^−1^ at 0 (top) and 24 h (bottom). Scale bars represent 100 µm. Scale bars in zoomed‐in images indicate 50 µm. The size distribution of PM2.5 detected in the brain side for the treatment of 0.1, 1, and 10 µg mL^−1^ of PM2.5 at 24 h was fitted to the normal distribution to determine the available size of PM2.5 passing through BBB (*n* = 5). d) Microglia infiltration in Tri‐PMB models. Microglia (MG, red) were initially presented in the annular chamber and observed in the central chamber of Tri‐PMB (marked as +PM) in 48 h in response to PM2.5‐driven inflammatory mediators, while no recruitment in that of Tri‐Con. Scale bars represent 1 mm. The bar graphs show the increasing number of microglia recruitment as the longer period of PM2.5 exposure (*n* = 4). Data represent means ± SD. *, *p* < 0.05; **, *p* < 0.01; measured by one‐way ANOVA with Bonferroni post‐hoc correction for multiple comparisons. e) Fluorescent images of Tri‐PMB representing the increased reactivity of astrocytes (top) and microglia (bottom) compared to Tri‐Con. Immunostaining of GFAP (top, yellow) and CD11b (bottom, yellow) was performed to evaluate the reactivity of astrocytes and microglia, respectively. Neural cells were stained with NeuN (green) and microglia were fluorescently labeled (red). Scale bars represent 50 µm. f,g) Quantitative results for GFAP and CD11b were summarized, respectively (*n* = 7). Data represent means ± SD. **, *p* < 0.01 and *f* > 0.8; measured by two‐tailed unpaired Student's *t*‐test for two variances. h) Assessment of PM2.5‐mediated neurodegeneration determined by the decrease in neuron population (*n* = 7). Data represent means ± SD. *, *p* < 0.05 and *f* > 0.8; measured by two‐tailed unpaired Student's *t*‐test for two variances. i) Assessment of PM2.5‐mediated neurodegeneration determined by LDH assay (*n* = 3). Data represent means ± SD. *, *p* < 0.05 and *f* > 0.8; measured by two‐tailed unpaired Student's *t*‐test for two variances.

Prior to testing potential risks of PM2.5 on the brain, we investigated whether PM2.5 could penetrate restrictive barriers around the human brain (**Figure** **1**a‐i). To explore the possibility of PM2.5 penetration, we tested PM2.5 transport across an in vitro paracellular barrier model, particularly the BBB model.^[^ ^10^ ^]^ We cultured human cerebral endothelial cells (hCMEC/D3) on the collagen‐I‐coated Trans‐well platform (Figure 1b‐i) and incubated them for 3 days of serum starvation to upregulate formation of tight junctions. We confirmed the formation of tight junctions in the BBB model by estimation of an apparent permeability of 40 kDa fluorescein isothiocyanate (FITC)–dextran (Pe = 1.0 × 10^−5^ cm s^−1^). Afterward, we added various concentrations of PM2.5 ranging from 0.1 to 10 µg mL^−1^ to the lumen side (upper chamber) and assessed the amount of PM2.5 precipitated on the brain side (bottom chamber) of Trans‐well plate. Optical microscopy detected notable aggregations forming on the bottom of the Trans‐wells during the 24 h observation period (Figure 1c, left panel) and the size of the aggregates was measured. Our results showed that treatment of PM2.5 at the concentrations of 0.1, 1, and 10 µg mL^−1^ resulted in the penetration of PM2.5 at the concentrations of 0.1, 0.1, and 100 ng mL^−1^ in 24 h and the formation of aggregates in the bottom of the Trans‐well plate in the average sizes of 0.0 ± 0.5, 3.1 ± 1.5, and 3.8 ± 2.0 µm (mean ± standard deviation (SD)), respectively (Figure 1c, right panel). These results confirmed that the PM2.5 can penetrate the in vitro BBB model. To investigate underlying mechanisms of PM2.5 increasing the permeability of BBB layer, we assessed the expression level of zonula occludens‐1 (ZO‐1), a gold standard tight junction marker, by immunostaining (Figure S1a, Supporting Information). We found that PM2.5 treatments significantly decreased the expression level of ZO‐1 in hCMEC/D3 cells, indicating that the increased BBB permeability was attributed to the downregulation of tight junctions by PM2.5. Furthermore, the viability test by lactate dehydrogenase (LDH) assay confirmed that the penetration of PM2.5 was not caused by cell death of endothelial cells on the Trans‐well inserts (Figure S1b, Supporting Information). Collectively, these results demonstrated that PM2.5 is capable of penetration into the brain endothelial layer by disrupting the tight junctions in the BBB.

### 2.2 Neuroinflammation in PM2.5‐Treated Human Brain Models

We recently developed a 3D human cellular brain model by triculturing neurons, astrocytes, and microglia in a chemotactic microfluidic platform that closely mimicked cellular interactions of neurons and innate immunity in human brains.^[^ ^11^ ^]^ In this study, we modified our versatile microfluidic platform and created a PM2.5‐polluted brain model with neurons, astrocytes, and microglia (Tri‐PMB, Figure 1b‐ii) to investigate the innate immune response toward PM2.5 microenvironments (Figure 1a‐ii). For control experiments, we employed our model without the PM2.5 treatment (Tri‐Con). Timeline for the model preparation was summarized in Figure 1b‐iii. Briefly, human neuroprogenitor cells (ReNcell VM) were cultured in the central chamber with 20% Matrigel. After 2 weeks of differentiation under serum starvation, we confirmed their differentiation into neurons and astrocytes by immunostaining of neuron‐specific class III beta‐tubulin (Tuj1) and glial fibrillary acidic protein (GFAP), respectively (Figure S2, Supporting Information). Through this method, the human neuroprogenitor cells were differentiated into ≈68% neurons (microtubule‐associated protein 2‐positive) and ≈30% astrocytes (GFAP‐positive).^[^ ^12^ ^]^ It should be noted that the populations for other type of cells were under 2%, which could be negligible. Upon completion of differentiation, we treated them with PM2.5 at 10 µg mL^−1^ (for Tri‐PMB) or without (for Tri‐Con) up to 9 weeks. Afterward, we loaded fluorescently labeled microglia in red color on the annular chamber, which was linked to the migration channels. The migration channels formed gradients of soluble factors from the central chamber, so that could recruit microglia into central chamber in 48 h (Figure 1d). We counted and plotted the recruited microglia in the central chamber as a function of PM2.5 treatment time. Our data indicated that more microglia were recruited in response to soluble factors from the longer they were exposed to PM2.5. Upon microglia recruitment, we assessed the reactivity in both astrocytes and microglia in the central chamber by immunostaining of GFAP and CD11b (Figure 1e). Our data showed that the GFAP (10.5‐fold) (Figure 1f) and CD11b (4.7‐fold) (Figure 1g) were significantly promoted in the Tri‐PMB compared to that in the Tri‐Con, representing the presence of reactive astrocytes and microglia in the Tri‐PMB. It should be noted that PM2.5 treatment on single‐cultured microglia did not increase the microglial reactivity, while that on cocultured neurons and astrocytes increased the astrocyte reactivity (Figure S3, Supporting Information). This would indicate that the cellular interactions between the neurons and astrocytes occurred initially and would further induce PM2.5‐driven neuroinflammation by recruiting and activating microglia in the brain. In addition, we observed significant reduction in the population of neurons (40.2%) (Figure 1h) and increased LDH level (1.7‐fold) (Figure 1i) in the Tri‐PMB, compared to the Tri‐Con, indicating the precipitation of neuronal damages in the Tri‐PMB. Overall, our Tri‐PMB represented the elevated neuroinflammation and neurodegeneration under PM2.5‐treated microenvironments.

### 2.3 Induction of Reactive Astrocytes in PM2.5‐Treated Human Brain Models

**Figure 2:**
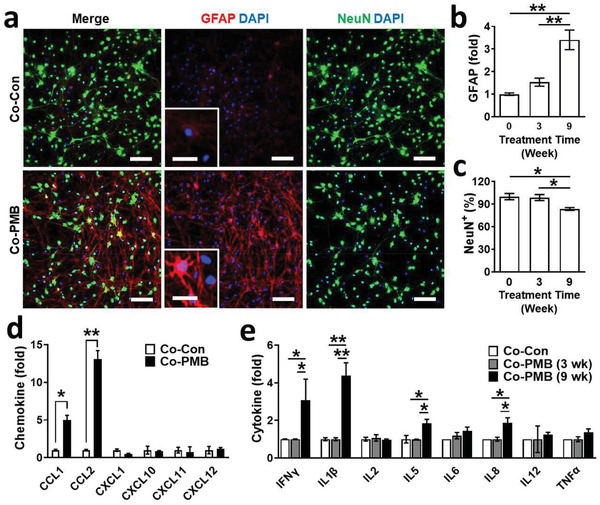
Induction of reactive astrocytes by PM2.5. a) Immunostaining results of Co‐Con and Co‐PMB for neurons (NeuN, green) and astrocytes (GFAP, red). Scale bars represent 50 µm. Zoomed‐in images focus on astrocyte‐positive cells. Scale bars represent 15 µm. b) Increased astrocyte reactivity was assessed by fluorescent intensity of GFAP as a function of PM2.5 treatment time in Co‐PMB (*n* = 7). Data represent means ± SD. **, *p* < 0.01; measured by one‐way ANOVA with Bonferroni post‐hoc correction for multiple comparisons. c) Decrease of neuronal population was measured by counting NeuN‐positive cells (*n* = 7). Data represent means ± SD. *, *p* < 0.05; measured by one‐way ANOVA with Bonferroni post‐hoc correction for multiple comparisons. d) Production of chemokines by Co‐Con or Co‐PMB (*n* = 4). Data represent means ± SD. *, *p* < 0.05; **, *p* < 0.01; measured by one‐way ANOVA with Tukey post‐hoc correction for multiple comparisons. e) Production of cytokines by Co‐Con or Co‐PMB (*n* = 4). Data represent means ± SD. *, *p* < 0.05; **, *p* < 0.01; measured by one‐way ANOVA with Tukey post‐hoc correction for multiple comparisons.

To further understand the underlying mechanisms of PM2.5‐driven neuroinflammation, we studied the initiation of neuroinflammation driven by interactions between neurons and astrocytes cocultured in the central chamber. Recent animal model studies have confirmed that inhalation of PM2.5 induced hyper‐reactivated astrocytes that elevated proinflammation (IL‐1*β*, IL‐6, TNF‐*α*, IFN‐*γ*, complements) in the brain that may consequently lead to neurodegeneration.^[^ ^6c,f^ ^]^ Thus, we investigated the induction of astrogliosis in the PM2.5‐treated conditions without microglia. To this end, we prepared cocultured models of neurons and astrocytes without (Co‐Con) and with PM2.5 treatment for 9 weeks (Co‐PMB). As we observed in the Tri‐PMB, the level of GFAP was significantly increased in Co‐PMB compared to Co‐Con, indicating the induction of reactive astrocytes in Co‐PMB (**Figure** **2**a,b). We observed the decrease in population of neuronal cells assessed by counting the NeuN‐positive cells in Co‐PMB (Figure 2a,c), presumably due to H_2_O_2_ (≈20 × 10^−6^ m), a source of oxidative stress produced by astrogliosis in response to PM2.5 (Figure S4, Supporting Information). These data are comparable to our previous results, whereby viability of neuronal cells was affected by oxidative stress produced by astrocytes.^[^ ^13^ ^]^ Next, we measured chemokines and cytokines produced by Co‐PMB with a multicytokine assay. The results showed that Co‐PMB increased the production of chemokine ligand 1 (CCL1, 5.0‐fold) and chemokine ligand 2 (CCL2, 13.0‐fold) (Figure 2d), well‐known chemokines to recruit microglia.^[^ ^11^ ^]^ In addition, we found that Co‐PMB induced significant levels of proinflammatory cytokines, particularly IFN‐*γ* (3.1‐fold) and IL‐1*β* (4.4‐fold) (Figure 2e), which may contribute to the polarization of M1 microglia promoting detrimental neuroinflammation. These data represented that microglia can further increase neuroinflammation in response to these soluble factors released from the cocultured neurons and astrocytes under PM2.5 conditions.

### 2.4 Migration of Microglia in Response to the Soluble Factors from PM2.5‐Treated Human Brain Models

**Figure 3:**
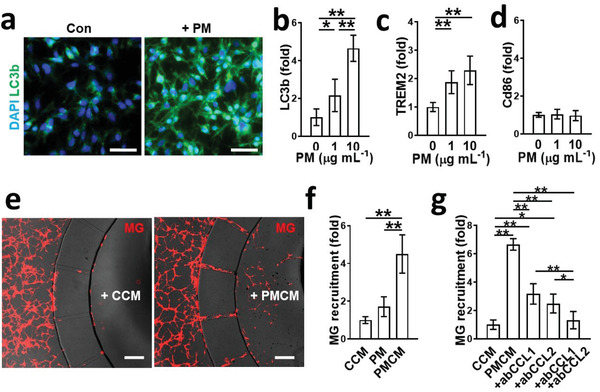
Microglia migration in response to soluble factors released by PM2.5‐treated neurons and astrocytes. a,b) Increased phagocytic activity of microglia in response to PM2.5. The phagocytic activity was assessed by immunostaining of LC3b. Scale bars represent 50 µm (*n* = 8). Data represent means ± SD. *, *p* < 0.05; ** *p* < 0.01; measured by one‐way ANOVA with Bonferroni post‐hoc correction for multiple comparisons. c) Activation of microglia serving neuroprotective roles with both sensing and phagocytic activity (TREM2‐positive) by PM2.5. *, *p* < 0.05 and *f* > 0.8 versus control (*n* = 8). d) No detectable transition of M1 type microglia (CD86‐positive) by PM2.5 (*n* = 8). Data represent means ± SD. **, *p* < 0.01; measured by one‐way ANOVA with Bonferroni post‐hoc correction for multiple comparisons. e) Notable number of microglia (red) were recruited to the central chamber with the conditioned media of Co‐PMB (PMCM) compared to that of Co‐Con (CCM). Scale bars represent 200 µm. f) Quantification of microglia migration. PMCM significantly increased microglia recruitment compared to CCM (*n* = 4). Data represent means ± SD. **, *p* < 0.01; measured by one‐way ANOVA with Bonferroni post‐hoc correction for multiple comparisons. g) Validation of CCL1 and CCL2 as key chemokines in PMCM to recruit microglia. The addition of blockage antibody either for CCL1 (ab CCL1, 1 µg mL^−1^) or CCL2 (ab CCL2, 1 µg mL^−1^) to PMCM significantly reduced the microglia recruitment compared to PMCM (*n* = 5). The addition of blockage antibodies for both CCL1 and CCL2 completely blocked the microglia recruitment (*n* = 5). Data represent means ± SD. *, *p* < 0.05; **, *p* < 0.01; measured by one‐way ANOVA with Bonferroni post‐hoc correction for multiple comparisons.

Since the microglia are primary cells infiltrating into the polluted area and providing immune surveillance,^[^ ^6a–e^ ^]^ we examined whether PM2.5 would affect sensing and phagocytic activities of microglia. To this end, we treated single‐cultured microglia with PM2.5 at various concentrations not affecting viability (Figure S3c, Supporting Information) and monitored the activity of microtubule‐associated protein light‐chain 3 (LC3)‐associated phagocytosis by immunostaining against LC3b, the active form of LC3 (**Figure** **3**a,b). We found that PM2.5 significantly promoted the level of LC3b in the microglia. In addition, microglia exposed to PM2.5 exhibited disease‐associated microglia (DAM) microglia phenotype (TREM2‐positive), which represented the activated microglia for phagocytosis serving neuroprotective roles (Figure 3c). However, the single‐cultured microglia did not retain M1 type in response to PM2.5 treatment alone (Figure 3d and Figure S3 (Supporting Information)).

We next hypothesized that the soluble factors released by cocultured damaged neurons and/or reactive astrocytes may initiate the recruitment of microglia, therefore enhancing PM2.5‐driven neuroinflammation. To test our hypothesis, we collected the conditioned media of Co‐PMB (PMCM) or Co‐Con (CCM) and added the conditioned media to the central chamber. Single‐cultured microglia were loaded in the annular chamber. Within 2 days, we observed significantly increased migration of microglia toward the PMCM‐treated chamber, while no migration toward the CCM‐treated chamber (Figure 3a). There was a slight increase in the number of microglia migrating toward PM2.5 only (Figure 3b), but it should be noted that single‐cultured microglia were not activated in response to PM2.5 treatment alone as shown previously (Figure S3, Supporting Information). Interestingly, PMCM supplemented with 1 µg mL^−1^ of CCL1 (ab CCL1) or CCL2 blocking antibody (ab CCL2) significantly reduced the PMCM‐mediated migration to the central chamber (Figure 3g). In addition, the blockage of both CCL1 and CCL2 completely blocked the migration, indicating the pivotal role of CCL1 and CCL2 in the recruitment of microglia. Taken together, these data indicated that microglia can be activated and recruited to the PM2.5‐polluted brain areas in response to chemokines such as CCL1 and CCL2 released by brain tissues rather than PM2.5 itself.

### 2.5 M1 Polarization in PM2.5‐Treated Human Brain Models

As the primary cells offering the innate immune surveillance, microglia are heterogeneous and can be activated into the proinflammatory state (M1) or the anti‐inflammatory state (M2) in response to contaminants, pathogens, and misfolded proteins.^[^ ^14^ ^]^ The M1 is a detrimental proinflammatory phenotype characterized by the elevated expression of markers (CD11b, CD86, inducible nitric oxide synthase (iNOS)), the production of inflammatory mediators (IL‐1*β*, TNF‐*α*, and IL‐6), and the release of neurotoxic NO.^[^ ^5^ ^]^ The M2 is a neuroprotective anti‐inflammatory phenotype characterized by the elevated expression of marker (CD206), anti‐inflammatory mediators (TGF‐*β*, IL‐10), growth factors insulin‐like growth factor‐1 (IGF‐1), fibroblast growth factor (FGF), colony stimulating factor‐1 (CSF‐1), and neurotrophic factors nerve growth factor (NGF), brain‐derived neurotrophic factor (BDNF), glial cell‐derived neurotrophic factor (GDNF).^[^ ^15^ ^]^ Recent studies revealed another unique microglial phenotype: DAM type, which is specialized for phagocytosis serving neuroprotective roles in response to air pollutants including PM2.5.^[^ ^16^, ^17^ ^]^ However, the microglial phenotypes in the PM‐polluted brains are still controversial due to the diversity and plasticity of microglial phenotypes in PM2.5‐polluted brains. In this study, we investigated how the soluble factors from neurons/reactive astrocytes could determine microglia phenotypes under PM2.5 conditions.

**Figure 4:**
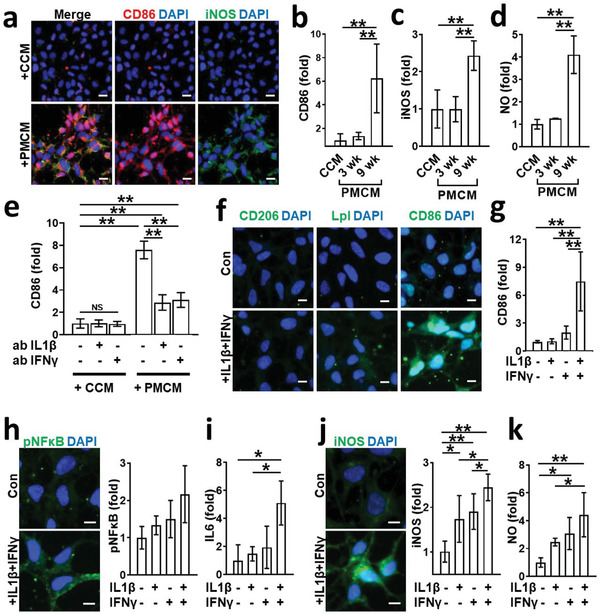
M1 polarization by soluble factors released from PM2.5‐treated neurons and astrocytes. a) Induction of M1 by PMCM. Microglia were exposed to either CCM (9 wk) or PMCM (9 wk) for 48 h and immunostained with M1 markers as CD86 (red) and iNOS (green). Scale bars represent 20 µm. b,c) Quantifications for CD86 and iNOS were represented as bar graphs, respectively (*n* = 7). Data represent means ± SD. **, *p* < 0.01; measured by one‐way ANOVA with Bonferroni post‐hoc correction for multiple comparisons. d) Assessment of NO production by microglia exposed to either CCM or PMCM for 48 h (*n* = 7). Data represent means ± SD. **, *p* < 0.01; measured by one‐way ANOVA with Bonferroni post‐hoc correction for multiple comparisons. e) Blockage of M1 transition by the addition of neutralizing antibody targeting either IL‐1*β* (ab IL‐1*β*, 100 ng mL^−1^) or IFN‐*γ* (ab IFN‐*γ*, 100 ng mL^−1^) to PMCM (*n* = 7). Data represent means ± SD. NS, no significance; **, *p* < 0.01; measured by one‐way ANOVA with Bonferroni post‐hoc correction for multiple comparisons. Conditioned media was supplemented with each neutralizing antibody and incubated for 1 h at room temperature prior to the treatment. f) Combined effects of IL‐1*β* and IFN‐*γ* on the M1 polarization. Microglia were treated with either control media or IL‐1*β* (10 ng mL^−1^) + IFN‐*γ* (10 ng mL^−1^) for 48 h and immunostained with microglial markers for M2 (CD206), DAM (Lpl), and M1 (CD86). Scale bars represent 10 µm. g) Quantifications for CD86 by immunostaining were represented as bar graphs (*n* = 8). Data represent means ± SD. ** *p* < 0.01; measured by measured by one‐way ANOVA with Bonferroni post‐hoc correction for multiple comparisons. h–k) Quantifications of pNF*κ*B by immunostaining (*n* = 8), IL‐6 by ELISA (*n* = 3), iNOS by immunostaining (*n* = 8), and NO by live cell imaging with NO probes (*n* = 7). Data represent means ± SD. *, *p* < 0.05; **, *p* < 0.01; measured by one‐way ANOVA with Bonferroni post‐hoc correction for multiple comparisons.

We treated microglia with either PMCM or CCM collected from cocultured models at 3 to 9 weeks for 48 h and investigated the polarization of proinflammatory microglia by immunostaining for CD86 (**Figure** **4**a,b). We set Co‐PMB models at 3 weeks to the early Co‐PMB models since we did not observe notable release of key proinflammatory cytokines such as IL‐1*β* and IFN‐*γ* (Figure 2e) as well as discernable neuronal death (Figure 2c). Our results showed that PMCM (9 wk) significantly increased the level of M1 marker (CD86, 6.2‐fold) compared to CCM (9 wk) (Figure 4a,b). However, PMCM (3 wk) increased DAM markers (Figure S5, Supporting Information) but did not promote the M1 marker. Given the fact that PMCM (3 wk) involved only PM2.5 not proinflammatory cytokines, we could conclude that PM2.5 itself did not promote M1 microglia. We next examined the production of NO by M1 microglia, which may increase oxidative stress and risk of neurotoxicity in the brain. The data showed that the treatment of PMCM (9 wk) promoted the activation of iNOS (2.4‐fold), the enzyme that is known to synthesize NO (Figure 4a,c). In addition, we found that PMCM (9 wk) treatment increased the production of NO (4.1‐fold) compared to that of CCM (9 wk) (Figure 4d), validating the production of free radicals by M1 microglia. It should be noted that PM2.5 treatment did not activate iNOS both in neurons and astrocytes (Figure S7, Supporting Information). Also, we did not detect any significant NO in CCM and PMCM. Taken together, the soluble factors released by PM2.5‐treated neurons and reactive astrocytes induced the polarization of M1 microglia producing neurotoxic factors.

Next, we investigated underlying mechanisms of M1 transition by PMCM. As shown in Figure 2e, PM2.5 treatment on cocultured neurons and astrocytes produced proinflammatory cytokines, with a majority comprising of IFN‐*γ* (≈10 ng mL^−1^) and IL‐1*β* (≈10 ng mL^−1^). It should be noted that other cytokines known to induce M1 microglia, such as IL‐6 and TNF‐*α* found in Co‐PMB were under 10 pg mL^−1^, which was not sufficient enough to induce M1 transition (Figure S6, Supporting Information). Similarly, Haghani et al. proved the accumulation of proinflammatory factors, such as IFN‐*γ* and IL‐1*β*, in brains of mice exposed via the respiratory tract to PM2.5.^[^ ^18^ ^]^ To demonstrate the contribution of IFN‐*γ* or IL‐1*β* on the M1 transition, we removed either IFN‐*γ* or IL‐1*β* from PMCM by using neutralizing antibody targeting IFN‐*γ* (ab IFN‐*γ*, 100 ng mL^−1^) or IL‐1*β* (ab IL‐1*β*, 100 ng mL^−1^). Interestingly, the addition of ab IFN‐*γ* or ab IL‐1*β* to PMCM significantly inhibited the M1 transition compared to PMCM without the neutralizing antibody (Figure 4e). To further understand the underlying mechanism of M1 transition either by IFN‐*γ* or by IL‐1*β*, we treated microglia with IFN‐*γ* (10 ng mL^−1^) and/or IL‐1*β* (10 ng mL^−1^). We observed that the combined treatment of IFN‐*γ* and IL‐1*β* notably upregulated the M1 polarization marker (CD86, 7.5‐fold), while single treatment of either IFN‐*γ* or IL‐1*β* did not (Figure 4f,g). However, the combined treatment did not change other phenotype markers, such as the M2 type (CD206) and the DAM type (lipoprotein lipase (Lpl)). To confirm the production of proinflammatory cytokine by M1 microglia, we also tested whether the combined treatment of IFN‐*γ* and IL‐1*β* increased the NF*κ*B activation (Figure 4h) and the IL‐6 production (Figure 4i). With increased M1 polarization, we found the additive effects of the combined treatment of IFN‐*γ* and IL‐1*β* in the NF*κ*B activation (2.2‐fold) and IL‐6 production (5.6‐fold). In addition, the combined treatment of IFN‐*γ* and IL‐1*β* increased iNOS (2.5‐fold) (Figure 4j) as well as NO (5.0‐fold) levels (Figure 4k). Taken together, our data suggest that both IFN‐*γ* and IL‐1*β* in PMCM were the major cytokines contributing to the polarization of M1 microglia, which led to the release of IL‐6 that may precipitate neuroinflammation and NO that may damage the brain.

**Figure 5:**
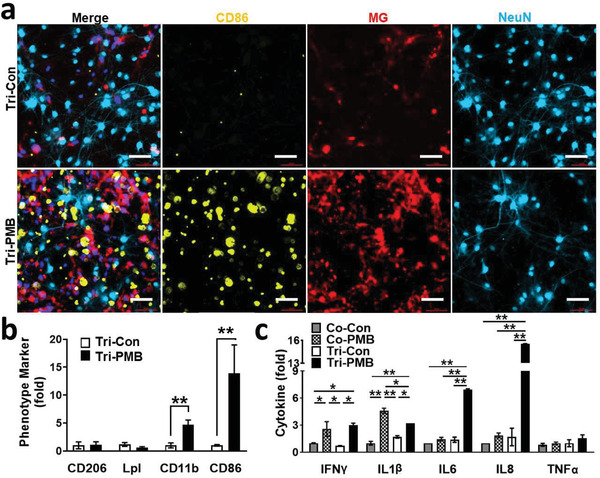
M1 polarization in Tri‐PMB model. a) Tri‐PMB model was immunostained with M1 marker (CD86, yellow) and Neu marker (NeuN, sky blue). Microglia were fluorescently labeled (red). Scale bars represent 50 µm. b) Quantitative results were represented as bar graphs (*n* = 7). Other phenotype markers, such as CD206, Lpl, and CD11b were also quantified after immunostaining and represented in (b) (*n* = 7). Data represent means ± SD. **, *p* < 0.01; measured by one‐way ANOVA with Bonferroni post‐hoc correction for multiple comparisons. c) Assessment of proinflammatory cytokines (*n* = 4). Data represent means ± SD. *, *p* < 0.05; **, *p* < 0.01; measured by one‐way ANOVA with Bonferroni post‐hoc correction for multiple comparisons.

We next demonstrated PM2.5‐driven microgliosis precipitating neuroinflammation in comparison between Co‐PMB and Tri‐PMB models. The immunostaining results demonstrated the polarization of M1 microglia in the Tri‐PMB, but no microglial activation in the Tri‐Con (**Figure** **5**a). Consistent with previous data set, PM2.5 treatment on our brain models increased M1 markers, such as CD11b (4.7‐fold) and CD86 (14.0‐fold). However, there was no significant change in markers representing other types of microglia, such as the M2 type (CD206, 1.1‐fold) and the DAM type (Lpl, 0.6‐fold) (Figure 5b), known to serve anti‐inflammatory and neuroprotective roles. We next explored the production of proinflammatory cytokines by M1 microglia in Tri‐PMB (Figure 5c). To subtract the proinflammatory mediators produced by neurons and astrocytes, we compared the results of tricultured model (Tri‐PMB) to that of cocultured neurons and astrocytes without microglia (Co‐PMB). The addition of microglia increased the production of IL‐6 (4.7‐fold) and IL‐8 (8.4‐fold) compared to the PM2.5‐treated condition without microglia. This indicated that M1 microglia in Tri‐PMB released proinflammatory cytokines, majorly IL‐6 and IL‐8. Taken together, our results validated the presence of M1 microglia serving proinflammatory roles in the Tri‐PMB.

### 2.6 Neurotoxic Proinflammation in PM2.5‐Treated Human Brain Models

**Figure 6:**
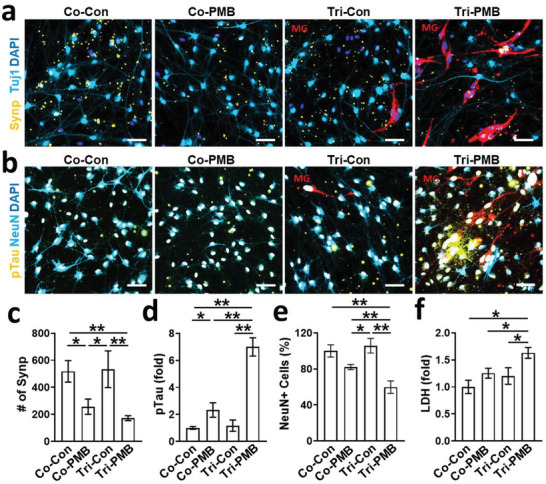
Neurodegeneration exacerbated by microgliosis in Tri‐PMB model. a) Precipitation of neuronal damages determined by the reduction of the presynaptic protein around the neurons. PMB models were immunostained with presynaptic protein marker (Synp, yellow) and neuronal marker (Tuj1, sky blue). Microglia were fluorescently labeled (red). Scale bars represent 50 µm. c) Quantitative results were represented as bar graphs (*n* = 4). Data represent means ± SD. *, *p* < 0.05; **, *p* < 0.01; measured by one‐way ANOVA with Bonferroni post‐hoc correction for multiple comparisons. b) Precipitation of neuronal damages determined by the accumulation of hyperphosphorylated tau in the neurons. PMB models were immunostained with hyperphosphorylated tau marker (pTau, yellow) and neuronal marker (NeuN, sky blue). Microglia were fluorescently labeled (red). Scale bars represent 50 µm. d,e) Quantitative results of pTau accumulation and NeuN reduction were represented as bar graphs, respectively (*n* = 7). Data represent means ± SD. *, *p* < 0.05; **, *p* < 0.01; measured by one‐way ANOVA with Bonferroni post‐hoc correction for multiple comparisons. f) Overall damages on PMB models were demonstrated by LDH assay (*n* = 3). Data represent means ± SD. *, *p* < 0.05; measured by one‐way ANOVA with Bonferroni post‐hoc correction for multiple comparisons.

To further investigate the neuronal damage induced by PM2.5‐driven neuroinflammation, we monitored changes in the expression level of synapsin I (Synp), which is present in the terminal of axons and regulates neurotransmission (**Figure** **6**a,c). Our data showed that the cocultured neurons and astrocytes with PM2.5 treatment (Co‐PMB) exhibited the reduction in the number of synapsin I by ≈49.6% compared to the nontreated cocultured model (Co‐Con). The synapsin I in Tri‐PMB was further decreased to 33.2% compared to the Co‐PMB. We next examined the implication of proinflammatory response on the accumulation of hyperphosphorylated tau (pTau) (Figure 6b,d), one of the major hallmarks involved in the progression of tauopathies and CNS disorders including AD, PD, and dementia.^[^ ^5^ ^]^ Our data showed that there was a slight increase in the models of PM2.5‐treated neurons and astrocytes (Co‐PBM) (2.3‐fold) compared to the nontreated group (Co‐Con). Interestingly, the addition of microglia to the PM2.5‐treated model (Tri‐PMB) notably increased the pTau precipitation, compared to Co‐Con/Tri‐Con (7.0‐fold) and Co‐PBM (3.0‐fold), indicating another key aspect of neurodegenerative microgliosis, which may increase the risk of CNS diseases. We indeed examined any amyloid response in PM2.5‐treated brain models, another risk factor associated with the tauopathy found in animal models exposed to air pollutants, which may promote AD progression.^[^ ^19^ ^]^ To this end, we measured soluble amyloid beta (A*β*) components such as A*β*1‐40 (A*β*40) and A*β*1‐42 (A*β*42) in 3D cultured Co‐Con and Co‐PMB models (Figure S8, Supporting Information). However, there was no detectable A*β*40 and A*β*42 in our PMB models. Finally, we explored the consequence of PM2.5‐driven neuroinflammation on the neuronal viability. To this end, we counted NeuN‐positive cells that represented the population for viable neuronal cells (Figure 6a,e). Our data showed that the population for neuronal cells was reduced to be 81.3% in the cocultured neurons and astrocytes treated with PM2.5 (Co‐PMB). Neuronal population was further reduced to be 59.7% by the addition of microglia, indicating that microglia played a neurodegenerative role in Tri‐PMB. We next measured the level of LDH as a mean of estimating the overall cytotoxicity in the models (Figure 6f). In accordance with the tendency of reduction in neuronal population, the data confirmed the increased cytotoxicity driven by PM2.5 (Co‐PMB, 1.2‐fold), which was further exacerbated by the addition of microglia (Tri‐PMB, 1.6‐fold). Overall, our data validated the neurodegenerative inflammation, which was exacerbated by microglia in the PM2.5‐treated brain models.

## 3 Discussion and Conclusions

Prominent activation of innate immune cells, such as astrocytes and microglia, followed by increase of neuroinflammation have been observed in PM2.5‐exposed brains.^[^ ^6a–e^ ^]^ However, the underlying mechanisms of their interplay under PM2.5 environments contributing to adverse neuroinflammation are extremely complex to be clarified with conventional animal models.^[^ ^9^ ^]^ Here, we employed our chemotactic microfluidic platform to engineer biomimetic models of PM2.5‐polluted human brains, which simplified but faithfully reproduced the interactive cellular interactions between neurons and glial cells. PM2.5 used in this study was obtained from diesel exhaust that involved PAHs and nitro‐PAHs inherently. This indicates that we examined potential risk of PM2.5 not only in the aspect of PM2.5 itself but also absorbed chemicals on PM2.5. By using our PMB models, we characterized PM2.5‐mediated astrogliosis and microgliosis separately, which have not been explored previously with classical animal models, human specimens, and other in vitro models.

First, we validated the translocation of PM2.5 to the brain region passing through the in vitro BBB model that may initiate neuroinflammation. Previously, Wang et al.reported that PM2.5 induced reactive‐oxygen‐species‐mediated calcium leakage in endothelial cells that attenuated the ZO‐1 level consequently while sparing other tight junctions, such as vascular endothelial‐cadherin and *β*‐catenin.^[^ ^20^ ^]^ In accordance with the previous study, we found that the treatment of PM2.5 decreased the ZO‐1 level but did not affect the cell viability of brain endothelial cells. Therefore, PM2.5 may disrupt the tight junctions between adjacent endothelial cells, penetrate the BBB (Figure 1b‐i), and may further promote neuroinflammation in the brain part (Figure 1b‐ii).

Second, this study elucidated sequences of PM2.5‐driven neuroinflammation driven by astrocytes followed by microglia. Astrocytes, well‐studied innate immune cells found in PM2.5‐inhaled animal brain models, acquired the hyper‐reactive state elevating sources of proinflammation and oxidative stress in the brains that may consequently activate other innate immune cells and increase brain inflammation.^[^ ^6c,f^ ^]^ Similarly, we found that astrocytes cocultured with neurons were activated by PM2.5 treatment, increasing the expression level of GFAP. In addition, cocultured PM2.5 models with the reactive astrocytes produced significant levels of proinflammatory chemokines (CCL1 and CCL2) and cytokines (IL‐1*β* and IFN‐*γ*), which can recruit and activate microglia. On the other hand, we did not detect any activation in the single‐cultured microglia under PM2.5 conditions. In this regard, we speculate that PM2.5‐driven neuroinflammation may be initiated by the reactive astrocytes cocultured with neurons rather than microglia.

Next, we explored how the PM2.5‐driven inflammatory soluble factors induced microgliosis. Our studies confirmed the notable recruitment of microglia in response to CCL1 and CCL2 in the conditioned media of injured neurons and reactive astrocytes exposed to PM2.5. In addition, we found the combined effects of IL‐1*β* and IFN‐*γ* from cocultured neurons and astrocytes under PM2.5 conditions on the transition of M1 microglia, producing NO and proinflammatory cytokines, such as IL‐6 and IL8. Several studies revealed the priming effects of IFN‐*γ* that sensitized microglia to other chemokines and cytokines and boosted proinflammation consequently.^[^ ^21^ ^]^ Moreover, IL‐1*β* increased the expression level of IFN‐*γ* receptors (IFN‐*γ*R1) on various innate immune cells, which contributed to the promotion of proinflammation in advance.^[^ ^22^ ^]^ Given the priming effects of IFN‐*γ* and IL‐1*β* on one another, we assume that IFN‐*γ* may increase the sensitivity of microglia toward IL‐1*β*, and vice versa, leading to the polarization of M1 microglia.

Finally, our studies concluded that air pollutants, specifically PM2.5, are risk factors for neurodegeneration due to increased neuroinflammation demonstrated by our experiments. A number of previous studies revealed that reactive astrocytes in the PM2.5‐polluted animal brains were involved in the promotion of brain damage.^[^ ^6c,f^ ^]^ In addition, microglia infiltrated into the polluted region and produced significant neurodegenerative sources increasing brain damage.^[^ ^6a–e^ ^]^ We disclosed that both astrogliosis and microgliosis increased the synaptic impairment in PMB models, while microgliosis dominantly contributed to neuronal death. We assume that neurotoxic NO from M1 microglia would be a major factor to decrease neuronal viability in accordance with our previous study.^[^ ^11^ ^]^ Furthermore, the PM2.5‐driven microgliosis promoted significant pTau accumulation in Tri‐PMB. We presume that IL‐6 from M1 microglia may contribute to the pTau accumulation as IL‐6 is a well‐known microglial proinflammatory mediator promoting tauopathy.^[^ ^23^ ^]^ Therefore, our data indicate that brains exposed to PM2.5 would have a high chance of experiencing pTau‐associated brain disorders, such as AD and PD. In our future study, we aim to clarify the roles of microglial NO and IL‐6 in the PMB models in terms of neurodegeneration and tauopathy.

In this study, we developed cell‐based PM2.5‐polluted brain models to investigate neuroinflammation induced by the chronic pulmonary exposure to diesel exhaust PM2.5. To be appropriate for short‐lifetime cell models, we adopt the concentrations ranging 1–10 µg mL^−1^, which are higher than in the ambient of heavy traffic regions (1 ng mL^−1^),^[^ ^24^ ^]^ but similar with other in vitro models.^[^ ^25^ ^]^ As a result, we could reduce model preparation time dramatically while maintaining the viability of endothelial cells and microglia (Figures S1b and S3c, Supporting Information). The neuronal damage in our PMB models were comparable to other in vitro models,^[^ ^26^ ^]^ but more severe than in vivo chronic models.^[^ ^27^ ^]^ First, we attributed the lower sensitivity toward PM2.5‐driven neuroinflammation to the heterogeneous populations of glial cells playing either proinflammatory or anti‐inflammatory roles depending on brain regions,^[^ ^6^, ^16^, ^17^ ^]^ while our models could separate PM2.5‐involving proinflammatory immune cells from other populations. Second, inhaled particles can be removed by other immune cells in the respiratory system, such as alveolar macrophages and lung lymphatics, before reaching to the brain,^[^ ^25^, ^28^ ^]^ while our cellular models are a type of an acute model as we employed larger amounts of PM2.5 as described in the previous response. In this regard, we believe that our model can represent the neurodegenerative features promoted by PM2.5 effectively.

Taken together, we, for the first time, have clarified the underlying mechanisms of neurodegeneration mediated by PM2.5‐driven neuroinflammation by using our novel multicellular platform that closely reproduces human brain tissues and cellular interactions of innate immunity in the brains. We unraveled underlying mechanisms of neuroinflammation driven by reactive astrocytes and M1 microglia, which may lead to the neurodegeneration found in the PM2.5‐exposed brains. This study offered critical and valuable information such as the significant impact of PM2.5 on neuroinflammation and transformative neuroscience aspects such as PM2.5‐driven other neurological disorders including AD, PD, and dementia. We believe that this study will contribute to our understanding how air pollutants could drive neuroinflammation and may further improve preventive or curable therapeutics for neurodegeneration. Furthermore, we envision that our brain models serve as robust and reliable in vitro platforms, which permit multiple high‐throughput analyses under controlled microenvironments and investigate the underlying mechanisms of other air‐pollutants‐driven neuroinflammation.

## 4 Experimental Section

### Preparation of PM2.5 Solution

Diesel particulate matter 2.5 (NIST SRM 1650b, National Institute of Standards and Technology, Gaithersburg, MD) stock solution (PM2.5 stock solution, 10 mg mL^−1^) was prepared in dimethyl sulfoxide (DMSO, Sigma‐Aldrich, St. Louis, MO) and sonicated for 30 min at room temperature (RT) to disperse the particles in the solution.^[^ ^29^ ^]^ Afterward, PM2.5 solution was diluted in Dulbecco's modified Eagle medium (DMEM)/F‐12 (Thermo Fisher Scientific, Waltham, MA)‐based differentiation media consisting of: 2 µg mL^−1^ of heparin (STEMCELL Technologies, Vancouver, Canada); 2% v/v B27 neuronal supplement (Thermo Fisher Scientific); and 1% v/v penicillin–streptomycin–amphotericin‐B solution (Lonza, Basel, Switzerland). The final concentrations were varied depending on experimental settings. For control counterparts, DMEM/F‐12 differentiation medium was employed including 0.1% DMSO, 2 µg mL^−1^ of heparin, 2% v/v B27, and 1% v/v penicillin–streptomycin–amphotericin‐B solution without PM2.5 component.

### Preparation of In Vitro BBB Models

The in vitro BBB models were prepared according to the method previously reported.^[^ ^10^ ^]^ Briefly, the insert of 24‐Trans‐well plate (Corning, Corning, NY) was coated with Collagen I (Corning) for 1 h so that endothelial cells could attach to the surface. Afterward, the insert cultured with 10 000 cells of hCMEC/D3 cells (Sigma‐Aldrich) in 100 µL of a EndoGRO‐MV complete culture media (Sigma‐Aldrich) was placed in the Trans‐well plate containing 500 µL of the same culture media in the bottom well. Once the cells reached to 90% of confluency (after ≈2 days), they were serum‐starved for 3 days by incubation in a serum‐free EndoGRO‐MV complete culture media to enhance the tight junction formation between adjacent endothelial cells and prevent multilayered paracellular barriers on the surface of Trans‐well insert. The BBB models were accepted if the effective permeability coefficient of a fluorescent‐labeled 40 kDa dextran (Thermo Fisher Scientific) was less than ≈1.0 × 10^−5^ cm s^−1^, which validated the reasonable integrity of BBB tight junctions. Details were described in the “Assessment of BBB Permeability” subsection.

### Assessment of BBB Permeability

To measure the permeability across the BBB model, 10 µg mL^−1^ of FITC–4 kDa dextran was added into the upper chamber (BBB lumen side). After 0 and 24 h of incubation, samples (100 µL) from both the top chamber and bottom chamber (brain side) of the Trans‐well plate were taken and the fluorescent intensity was measured using a microplate reader (Synergy HT, BioTek Instruments, Winooski, VT). The apparent permeability of each sample across the BBB (*P*_BBB_) was calculated by Equation (1)^[^ ^30^ ^]^

(1) $$P_{\mathrm{BBB}}\;=\;\frac{C_{L}}{C_{0}}\cdot \frac{V}{S\cdot t}$$

where *P*_BBB_ represents the permeability coefficient of the dextran across the BBB (cm s^−1^), *C*_L_ is the concentration of the dextran at 24 h in the bottom chamber (µg mL^−1^), *C*_0_ is the initial concentration of the dextran at 0 h in the top chamber (µg mL^−1^), *V* is the volume of bottom chamber (cm^3^), *S* is the surface area of BBB layer (cm^2^), and *t* is the incubation time (s). The BBB models relevant were assessed because the measured permeability coefficient was 1.0 × 10^−5^ cm s^−1^ for 40 kDa dextran, which was close to 4 × 10^−5^ cm s^−1^ in previously validated BBB models.^[^ ^31^ ^]^

### Chip Fabrication

The chemotactic chip was employed to create a PM2.5‐polluted brain model. Details of the chip design were described in the previous study.^[^ ^32^ ^]^ To fabricate a mold of the devices, a SU‐8 negative photoresist (MicroChem, Round Rock, TX) was sequentially patterned using photolithography on a silicon wafer. A mixture of base and curing agent of Sylgard 184 A/B polydimethylsiloxane (PDMS) (Dow Corning, Midland, MI) was poured onto the SU‐8 mold to replicate the microstructures. The cured PDMS replica was removed from the mold, and holes were created for fluid reservoirs. Plastic chambers for media reservoirs were fabricated with a computer‐controlled Zing laser cutter (Epilog Laser, Golden, CO) with a 6 mm thick acrylic plate. The replicated PDMS and plastic layers were glued together using PDMS. The resultant assembly was irreversibly bonded to a customized glass‐bottomed uniwell plate (MatTek, Ashland, MA) by oxygen plasma treatment (Plasma Etch, Carson City, NV). Prior to the cell culture on the device, each chamber was coated with 1% v/v Matrigel matrix (Corning) diluted in DMEM/F‐12 for 1 h and washed with phosphate‐buffered saline (PBS) thoroughly.

### Preparation of Human PM2.5‐Polluted Brain Models

The 3D human PM2.5‐polluted model was utilized to investigate the adverse effects of PM2.5‐driven neuroinflammation on human brains (Figure 1b‐iii). Two weeks before the PM2.5 treatment (−W2), human neural progenitor cells (ReNcell VM, Sigma‐Aldrich) were detached from the culture plate and harvested in 100 µL of the differentiation media at the density of 1 × 10^7^ cells mL^−1^. Next, the cell solution was mixed with Matrigel in 1:5 ratio v/v; and 10 µL of the mixture was added to the central chamber of the microfluidic device to achieve 3D cultured ReN cells in 20% Matrigel. Additional 100 µL of the differentiation media was added to both the central chamber and two annular chambers per device. The microfluidic devices were placed in a 5% CO_2_ cell culture incubator at 37 °C. One‐half volume of the differentiation media in the central chamber was replaced every 3.5 days until the progenitor cells were fully differentiated into neurons and astrocytes (≈2 weeks). The replacement of one half of media every 3.5 days was designed to prevent any unexpected cell damage, caused by the shortage of nutrients in the 3D cultured models. From week 0 (W0), the central chamber media was replaced to 100 µL of PM2.5 diluent prepared in the differentiation media (PM2.5 solution, 10 µg mL^−1^). The PM2.5 concentration was adopted based on the preliminary studies not affecting cell viability of endothelial cells and microglia (Figures S1b and S3c, Supporting Information) but promoting neuroinflammation. It should be noted that the optimized treatment concentration (10 µg mL^−1^) was in the range that was tested in other in vitro models.^[^ ^26^ ^]^ From second treatment, one half of control or PM2.5 diluent was replaced every 3.5 days. For the control counterpart, the central chamber media was replaced to 100 µL of the differentiation media without PM2.5. At week 9 (W9), SV40 human adult microglia (Applied Biological Materials, Richmond, Canada) were detached from the cell culture plate and added to the annular chamber in the differentiation media supplemented with 2% v/v fetal bovine serum (FBS, Life Technologies) at the cell seeding density of 5000 cells per device. To avoid unexpected chemotaxis caused by FBS, the central chamber media was also replaced to the same media added to the annular chamber. The microfluidic devices were incubated in a 5% CO_2_ cell culture incubator at 37 °C for 2 days to complete microglia migration in response to the soluble factors from the central chamber.

### Migration Study

Upon the addition of microglia to the annular chamber, the number of recruited microglia was monitored in the central chamber for 2 days under the fully automated fluorescence microscope (Nikon TiE microscope, Nikon, Melville, NY). For the single‐culture study of microglia to investigate the underlying mechanism of microglia chemotaxis, microglia were loaded in the annular chamber while either CCM, PMCM, PM2.5 solution (10 µg mL^−1^), or PMCM supplemented with CCL2 blocking antibody (1 µg mL^−1^) were added to the central chamber. After 2 days of incubation in a 5% CO_2_ cell culture incubator at 37 °C, the number of microglia migrating to the central chamber was counted.

### Multicytokine Assay

Upon the completion of microglia migration to the central chamber, 1 mL of each conditioned media of control or PMB models were collected and employed for one data point. The instructions provided by the manufacturer were followed to assess chemokines and cytokines released by control and PMB models by using an ARY005B human cytokine array kit (R&D systems, Minneapolis, MN). The chemiluminescence signals were detected by using a ChemiDoc Imaging System (Bio‐Rad, Hercules, CA).

### Enzyme‐Linked Immunosorbent Assay (ELISA)

100 µL of each conditioned media of control or PMB models were collected and employed for one data point. The instructions provided by the manufacturer were followed to assess the levels of IL‐1*β*, IFN‐*γ*, and IL‐6 in the conditioned media, by using Human IL‐1*β* ELISA kit (BioLegend, San Diego, CA), Human IL‐IFN‐*γ* ELISA kit (Invitrogen), and Human IL‐6 ELISA kit (Invitrogen), respectively. The absorbances at 450 nm (primary signals) and 650 nm (reference signals) were assessed using a microplate reader (Synergy HT, BioTek Instruments, Winooski, VT).

### NO Measurement

Fold changes in NO concentration in microglia were monitored using the fluorescent NO probe, difluorofluorescein‐FM diacetate (DAF‐FM DA, Thermo Fisher Scientific). Briefly, cells were washed with PBS and treated with 5 × 10^−6^ m DAF‐FM DA diluted in the differentiation media supplemented with 5% v/v FBS for 30 min at 37 °C. Microglia were rinsed with PBS 3 times to remove excessive probes, added the fresh differentiation media supplemented with 5% v/v FBS, and incubated for an additional 30 min to complete the de‐esterification of diacetates in the cells. Afterward, NO was detected by using a fluorescence microscope (Nikon TiE microscope, Nikon) equipped with a FITC filter.

### Immunocytochemistry

For immunostaining of 3D cultured cells in the device, the models were rinsed with PBS twice and fixed with 4% paraformaldehyde (Electron Microscopy Sciences, Hatfield, PA) for 30 min at RT. Cells were then rinsed with PBS 2 times with 10 min intervals and incubated in the permeabilizing solution, PBS solution supplemented with 0.1% v/v Triton X‐100 and 0.1% v/v Tween 20, for 30 min at RT. Cells were next washed with PBS 3 times with 10 min intervals and incubated in the blocking solution, PBS solution supplemented with 0.1% v/v Tween 20 and 3% v/v human serum albumin, for 2 h at RT. Cells were again washed with PBS 3 times with 10 min intervals and incubated with the primary antibody diluted in the blocking solution. Details of primary and secondary antibodies in terms of dilution ratio and other information were summarized in Table S1 (Supporting Information). After secondary antibody reaction, the devices were washed 7 times with PBS supplemented with 0.1% v/v Tween 20 with 10 min intervals and examined under a fluorescence microscope (Nikon TiE microscope, Nikon). The intensity of immunoreactivity was analyzed by using a NIS‐Elements software.

### In Vitro Toxicology Assay

To assess the cytotoxicity of PM2.5‐driven neuroinflammation on the brains, LDH‐based toxicology assay was employed. Briefly, 100 µL of each conditioned media of control or PMB models were collected for one data point, and then the conditioned media was mixed with 100 µL of LDH assay reaction mixture (Sigma). After 1 h incubation at room temperature, the stop buffer was added to block the enzyme reaction and the signals at 490 nm were measured by using a microplate reader (Synergy HT, BioTek Instruments).

### Statistical Analysis

Data in graphs were presented as mean ± SD unless otherwise stated. The number of samples examined per group was specified for each set of data in the corresponding figure caption. Statistical significance for comparison of two experimental groups was determined by unpaired two‐tailed *t*‐test using GraphPad Prism 6 (GraphPad Software, La Jolla, CA). The probability value of *p* < 0.05 was considered significant. To further validate the significance, effect size was calculated by Cohen's D. The effect size of *f* > 0.8 was considered a large effect. Statistical significance for multiple comparisons was carried out by one‐way analysis of variance (ANOVA) with Bonferroni or Tukey post‐hoc correction using IBM SPSS Statistics Premium 27 (SPSS Inc., Chicago, IL).The probability value of *p* < 0.05 was considered significant. All statistical data were summarized in Table S2 (Supporting Information). The NS, *, and ** represented no significance, *p* > 0.05, and *p* < 0.01, respectively.

## Conflict of Interest

The authors declare no conflict of interest.

## Supporting information

Supporting Information

## Data Availability

Research data are not shared.
