# Supplementary material for: An Air Particulate Pollutant Induces Neuroinflammation and Neurodegeneration in Human Brain Models
Source: Adv Sci (Weinh). 2021 Sep 24;8(21):2101251. doi: 10.1002/advs.202101251 (PMC8564420; doi:10.1002/advs.202101251)
Supplement: Supplementary file 1 — Supporting Information [file ADVS-8-2101251-s001.pdf]

© 2021 Wiley-VCH GmbH

ADVANCED SCIENCE
Open Access

Supporting Information

for *Adv. Sci.*, DOI: 10.1002/advs.202101251

Air Particulate Pollutant Induces Neuroinflammation and Neurodegeneration in Human Brain Models

*You Jung Kang, Hsih-Yin Tan, Charles Y. Lee, and Hansang Cho**

Supporting Information

Title: Air particulate pollutant induces neuroinflammation and neurodegeneration in human brain models

*You Jung Kang, Hsih-Yin Tan, Charles Y. Lee, and Hansang Cho**

Effects of PM2.5 on in vitro BBB models: We investigated underlying mechanisms of PM2.5 penetration into *in vitro BBB models*. Figure S1a showed that PM2.5 treatment to BBB models downregulated the level of tight junction (ZO-1) in hCMEC/D3 cells significantly, which may decrease the integrity of BBB tightness and increase BBB penetration. Figure S1b confirmed that the increase of BBB penetration was not attributed to the cell death by PM2.5 treatment.

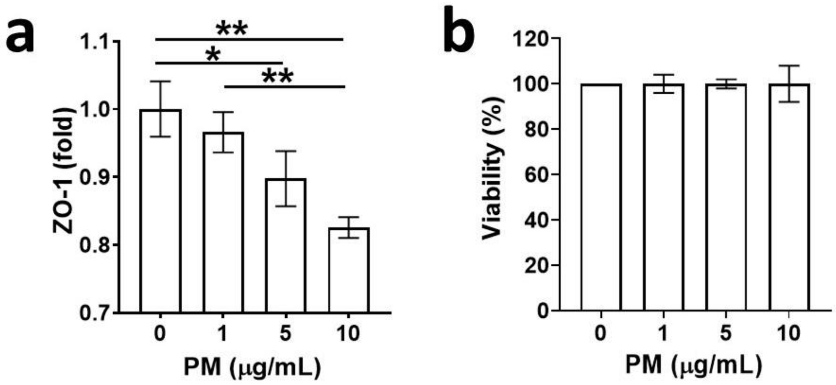

Figure S1. Effects of PM2.5 on the *in vitro* BBB. (a) Decrease of ZO-1 in hCMEC/D3 cells by PM2.5. *In vitro* BBB models with hCMEC/D3 cells were treated with various concentrations of PM2.5 (0 to 10 µg mL^{-1}) for 24 hrs and immunostained with ZO-1 (n=8). Data represent means ± SD. *, $p<0.05$; **, $p<0.01$; measured by One-way ANOVA with Bonferroni post-hoc correction for multiple comparisons. (b) No detectable changes in viability of in hCMEC/D3 cells by the treatment of PM2.5. The viability of hCMEC/D3 cells treated with various concentrations of PM2.5 (0 to 10 µg mL^{-1}) for 24 hrs was assessed by LDH assay (n=4).

Validation of differentiated neurons and astrocytes: Prior to PM2.5 treatment, we confirmed the differentiation of neural progenitor cells into neurons and astrocytes by immunostaining with Tuj1 and GFAP, respectively. Figure S2 showed the presence of both neurons and astrocytes in the models.

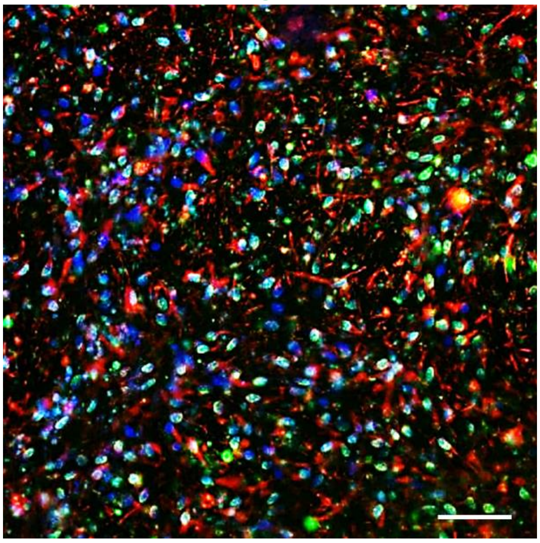

Figure S2. Immunofluorescent micrograph validated the differentiated neurons with Tuj1 (green) and astrocytes with GFAP (red). Nucleus was stained by DAPI (blue). Scale bar represents 50 µm.

Assessment of glial reactivity in response to PM2.5: We checked whether PM2.5 could increase glial reactivity by immunostaining of Co-Con (co-cultured neurons and astrocytes) and SV40 Con (single-cultured microglia) models with astrocyte reactive marker (GFAP) and microglia reactive marker (CD11b). Figure S3 showed that PM2.5 treatment increased the reactivity of astrocytes while did not promoted the reactivity of microglia.

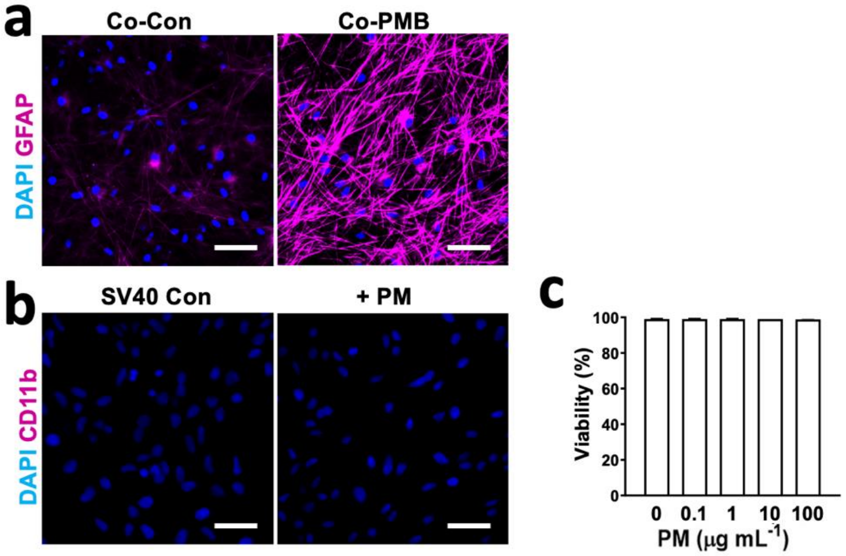

Figure S3. Effects of PM2.5 on the glial reactivity. (a) Increase of astrocyte reactivity by PM2.5 treatment. C-cultured models with (Co-PMB) or without PM2.5 (Co-Con) were immunostained with GFAP (pink) and DAPI (blue). Scale bars represent 50 µm. (b) No change in microglia reactivity by PM2.5 treatment. Single-cultured microglia with or without PM2.5 were immunostained with CD11b (pink) and DAPI (blue). Scale bars represent 50 µm. (c) No significant effect of PM2.5 on the microglia viability.

Production of a source of oxidative stress by Co-PMB: We validated the notable elevated level of H_2O_2 in PMCM (Figure S4), which is known to increase neuronal death in brain models.[13]

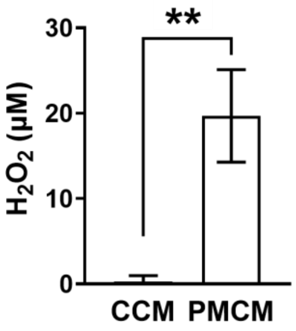

Figure S4. Measurement of H_2O_2 in condition media of Co-Con (CCM) and Co-PMB (PMCM) by using Amplex™ Ultra. There was a significant increase in the level of H_2O_2 in PMCM (19.691 ± 5.422 μM) compared to CCM (0.242 ± 0.736 μM) (n=3). Data represent means ± SD. **, $p<0.01$ and $f>0.8$; measured by two-tailed unpaired Student's t-test for two variances.

DAM transition in the early PMB models: We observed that DAM microglia were activated by PM2.5. We further investigate microglia response in the early PMB models (3 week) as there were only PM2.5 not key proinflammatory cytokines (IL1β and IFNγ) (Figure 2e). When we added microglia to the conditioned media of the early PMB model (PMCM (3 wk)), we found that microglia expressed DAM markers such as TREM2 and Lpl (Figure S5).

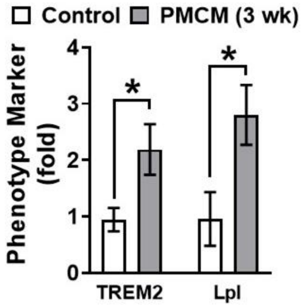

Figure S5. Transition of DAM type by PMCM (3 wk) (n=8). Data represent means ± SD. *, $p<0.05$ and $f>0.8$; measured by two-tailed unpaired Student's t-test for two variances.

Effect of IL6 and TNFα on M1 transition: We assessed proinflammatory cytokines produced by Co-PMB models. Among the cytokines, we found that the levels of IL1β and IFNγ were around 10 ng mL^{-1} while IL6 and TNFα were under 10 pg mL^{-1} representing that IL1β and IFNγ would majorly contribute to M1 transition. To confirm this, we have shown the combined effects of IL1β and IFNγ (Figure 4) on the M1 transition. On the other hand, we did not see any significant M1 transition by either IL6 or TNFα up to 10 ng mL^{-1} (Figure S6).

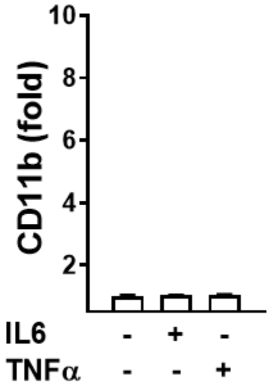

Figure S6. No significant M1 transition was induced by either IL6 or TNFα.

Study of iNOS in astrocytes in response to PM2.5: We examined whether PM2.5 could upregulate iNOS in astrocytes by immunostaining of Co-Con and Co-PMB models with antibodies targeting iNOS along with markers targeting astrocytes (GFAP) and neurons (NeuN). Figure S7 showed that PM2.5 treatment for 9 weeks did not increase the expression level of iNOS.

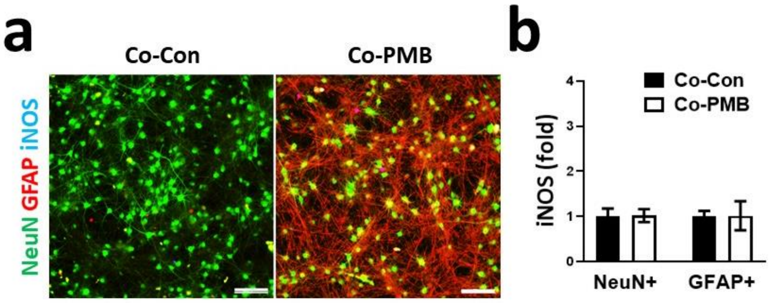

Figure S7. Effects of PM2.5 on the iNOS expression in neurons and astrocytes. (a) Fluorescent images represented neurons stained by NeuN (green) and astrocytes by GFAP (red). Both neurons and astrocytes did not activate iNOS (blue). Scale bars represent 100 µm. (b) There was no significant activation of iNOS both in neurons ($NeuN^+$) and astrocytes ($GFAP^+$) in response to PM2.5 (n=8).

Investigation of amyloid response in PMB models: We investigated whether PM2.5 promoted the production of amyloid beta (Aβ) in PMB models by measuring soluble Aβ1-40 (Aβ40) and Aβ1-42 (Aβ42). Figure S8 revealed that there was no detectable amyloid response in our PMB models.

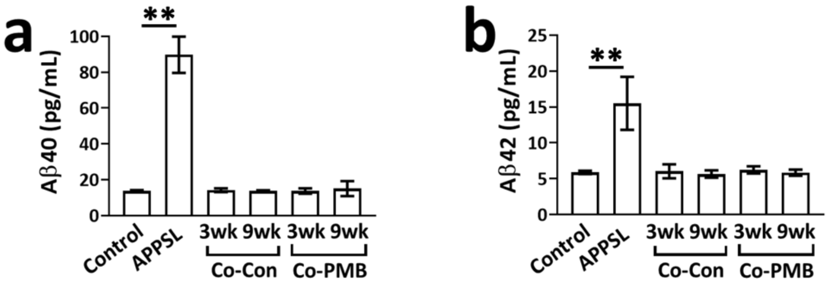

Figure S8. Measurement of (a) Aβ1-40 (Aβ40) and (b) Aβ1-42 (Aβ42) in Co-Con and Co-PMB models. For the positive control, we measured Aβ40 and Aβ42 in Co-Con expressing APPSL. There was no detectable amyloid response both in Co-Con and Co-PMB models while significant production of Aβ40 (89.7 ± 10.1 pg mL^{-1}) and Aβ42 (15.5 ± 3.7 pg mL^{-1}) in APPSL models (n=4). Data represent means ± SD. **, $p<0.01$; measured by One-way ANOVA with Bonferroni post-hoc correction for multiple comparisons.

Table S1. Antibodies used in the study

Antibodies	Company	Catalog #	Dilution ratio
ZO-1	Invitrogen	339100	1:50
GFAP	Sigma-Aldrich	AB5541	1:200
NeuN	Abcam	Ab177487	1:100
CD11b	Sigma-Aldrich	MABF515	1:100
CD86	Abcam	Ab196565	1:100
iNOS	Thermo Fisher Scientific	PA1-036	1:100
pNFκB	Abcam	Ab32536	1:100
CD206	Novus Biologicals	NB6001415	1:100
Lpl	Abcam	Ab21356	1:100
Tuj1	BioLegend	801210	1:100
Synapsin-1	Abcam	Ab254349	1:100
pTau (AT8)	Thermo Fisher Scientific	MN1020	1:100
LC3b	Abcam	Ab51520	1:100
TREM2	R&D System	AF1828	1:100
Goat anti-chicken 2’ ab	R&D System	NL017	1:200
Goat anti-mouse 2’ ab<br>Alexa 488<br>Alexa 555<br>Alexa 647	Abcam	A32723<br>A32727<br>A32728	1:200
Goat anti-rabbit 2’ ab<br>Alexa 488<br>Alexa 555<br>Alexa 647	Abcam	A32731<br>A32732<br>A32733	1:200

Table S2. Summary of statistical analysis

	Comparison	Sample number	Method(1)	P value	Significance(2)
Figure 1d	9 wk vs 0 wk	4	One-way ANOVA (Bonferroni post-hoc)	<0.001	Y
	3 wk vs 0 wk	4		<0.001	Y
	9 wk vs 3 wk	4		<0.001	Y
Figure 1f	Tri-PBM vs Tri-Con	7	t-test	<0.001	Y
Figure 1g	Tri-PBM vs Tri-Con	7	t-test	<0.001	Y
Figure 1h	Tri-PBM vs Tri-Con	7	t-test	0.0017	Y
Figure 1i	Tri-PBM vs Tri-Con	3	t-test	0.0030	Y
Figure 2b	9 wk vs 0 wk	7	One-way ANOVA (Bonferroni post-hoc)	<0.001	Y
	3 wk vs 0 wk	7		<0.001	Y
	9 wk vs 3 wk	7		<0.001	Y
Figure 2c	9 wk vs 0 wk	7	One-way ANOVA (Bonferroni post-hoc)	0.004	Y
	9 wk vs 3 wk	7		0.005	Y
Figure 2d	CCL1: Co-PMB vs Co-Con	4	One-way ANOVA (Tukey post-hoc)	0.012	Y
	CCL2: Co-PMB vs Co-Con	4		<0.001	Y
Figure 2e	IFNγ: Co-PMB (9 wk) vs Co-Con	4	One-way ANOVA (Tukey post-hoc)	0.028	Y
	IFNγ: Co-PMB (9 wk) vs Co-PMB (3 wk)	4		0.037	Y
	IL1β: Co-PMB(9 wk) vs Co-Con	4		<0.001	Y
	IL1β: Co-PMB(9 wk) vs Co-PMB (3 wk)	4		<0.001	Y
	IL5: Co-PMB (9 wk) vs Co-Con	4		0.029	Y
	IL5: Co-PMB (9 wk) vs Co-PMB (3 wk)	4		0.028	Y
	IL8: Co-PMB (9 wk) vs Co-Con	4		0.006	Y
	IL8: Co-PMB (9 wk) vs Co-PMB (3 wk)	4		0.008	Y
Figure 3b	10 μg mL^{-1} vs 0 μg mL^{-1}	8	One-way ANOVA (Bonferroni post-hoc)	<0.001	Y
	10 μg mL^{-1} vs 1 μg mL^{-1}	8		<0.001	Y
	1 μg mL^{-1} vs 0 μg mL^{-1}	8		0.008	Y
Figure 3c	10 μg mL^{-1} vs 0 μg mL^{-1}	8	One-way ANOVA (Bonferroni post-hoc)	<0.001	Y
	1 μg mL^{-1} vs 0 μg mL^{-1}	8		<0.001	Y
Figure 3f	PMCM vs CCM	4	One-way ANOVA (Bonferroni post-hoc)	<0.001	Y
	PMCM vs PM	4		<0.001	Y
Figure 3g	PMCM vs CCM	5	One-way ANOVA (Bonferroni post-hoc)	<0.001	Y
	+abCCL1 vs CCM	5		<0.001	Y
	+abCCL2 vs CCM	5		0.005	Y
	PMCM vs +abCCL1	5		0.004	Y
	PMCM vs +abCCL2	5		<0.001	Y
	PMCM vs +abCCL1/CCL2	5		<0.001	Y
	+ab CCL1 vs +abCCL1/CCL2	5		<0.001	Y
	+ab CCL2 vs +abCCL1/CCL2	5		0.040	Y
Figure 4b	9 wk vs 0 wk	7	One-way ANOVA (Bonferroni post-hoc)	<0.001	Y
	9 wk vs 3 wk	7		<0.001	Y
Figure 4c	9 wk vs 0 wk	7	One-way ANOVA (Bonferroni post-hoc)	<0.001	Y
	9 wk vs 3 wk	7		<0.001	Y
Figure 4d	9 wk vs 0 wk	7	One-way ANOVA (Bonferroni post-hoc)	<0.001	Y
	9 wk vs 3 wk	7		<0.001	Y
Figure 4e	PMCM vs CCM	7	One-way ANOVA (Bonferroni post-hoc)	<0.001	Y
	PMCM+abIL1β vs CCM	7		<0.001	Y
	PMCM +abIFNγ vs CCM	7		<0.001	Y
	PMCM vs PMCM+abIL1β	7		<0.001	Y
	PMCM vs PMCM +abIFNγ	7		<0.001	Y
Figure 4g	IL1β+IFNγ vs Control	8	One-way ANOVA (Bonferroni post-hoc)	<0.001	Y
	IL1β+IFNγ vs IFNγ	8		<0.001	Y
	IL1β+IFNγ vs IL1β	8		<0.001	Y
Figure 4i	IL1β+IFNγ vs Control	3	One-way ANOVA (Bonferroni post-hoc)	0.023	Y
	IL1β+IFNγ vs IL1β	3		0.044	Y
Figure 4j	IL1β+IFNγ vs Control	8	One-way ANOVA (Bonferroni post-hoc)	<0.001	Y
	IL1β vs Control	8		0.006	Y
	IFNγ vs Control	8		<0.001	Y
	IL1β+IFNγ vs IFNγ	8		0.027	Y
	IL1β+IFNγ vs IL1β	8		0.003	Y
Figure 4k	IL1β+IFNγ vs Control	7	One-way ANOVA (Bonferroni post-hoc)	<0.001	Y
	IL1β vs Control	7		0.073	Y
	IL1β+IFNγ vs IL1β	7		0.008	Y
Figure 5b	CD11b: Tri-PMB vs Tri-Con	7	One-way ANOVA	<0.001	Y

	CD86: Tri-PMB vs Tri-Con	7	(Bonferroni post-hoc)	<0.001	Y
Figure 5c	IFNγ: Tri-PMB vs Co-Con	4	One-way ANOVA (Bonferroni post-hoc)	0.013	Y
	IFNγ: Tri-PMB vs Tri-Con	4		0.011	Y
	IFNγ: Tri-Con vs Co-PMB	4		0.025	Y
	IFNγ: Co-PMB vs Co-Con	4		0.036	Y
	IL1β: Tri-PMB vs Co-Con	4		<0.001	Y
	IL1β: Tri-PMB vs Tri-Con	4		0.003	Y
	IL1β: Tri-PMB vs Co-PMB	4		0.004	Y
	IL1β: Tri-Con vs Co-PMB	4		<0.001	Y
	IL1β: Co-PMB vs Co-Con	4		<0.001	Y
	IL6: Tri-PMB vs Co-Con	4		<0.001	Y
	IL6: Tri-PMB vs Tri-Con	4		<0.001	Y
	IL6: Tri-PMB vs Co-PMB	4		<0.001	Y
	IL8: Tri-PMB vs Co-Con	4		<0.001	Y
	IL8: Tri-PMB vs Tri-Con	4		<0.001	Y
	IL8: Tri-PMB vs Co-PMB	4		<0.001	Y
Figure 6c	Tri-PMB vs Co-PMB	4	One-way ANOVA (Bonferroni post-hoc)	<0.001	Y
	Tri-PMB vs Tri-Con	4		<0.001	Y
	Tri-Con vs Co-PMB	4		0.003	Y
	Co-PMB vs Co-Con	4		0.005	Y
Figure 6d	Tri-PMB vs Co-PMB	7	One-way ANOVA (Bonferroni post-hoc)	<0.001	Y
	Tri-PMB vs Tri-Con	7		<0.001	Y
	Tri-PMB vs Co-Con	7		<0.001	Y
	Co-PMB vs Co-Con	7		0.035	Y
Figure 6e	Tri-PMB vs Co-PMB	7	One-way ANOVA (Bonferroni post-hoc)	<0.001	Y
	Tri-PMB vs Tri-Con	7		<0.001	Y
	Tri-PMB vs Co-Con	7		<0.001	Y
	Tri-Con vs Co-PMB	7		0.012	Y
Figure 6f	Tri-PMB vs Co-PMB	3	One-way ANOVA (Bonferroni post-hoc)	0.001	Y
	Tri-PMB vs Tri-Con	3		0.013	Y
	Tri-PMB vs Co-Con	3		0.030	Y

(1) One-way ANOVA was performed with either Bonferroni or Tukey post-hoc correction for multiple comparisons.
Two-tailed unpaired Student's t-test was performed for two variances.
(2) P value < 0.05 represents significance.

Table S3. Summary of PMB models employed in this study

	Central Chamber		Angular Chamber	PM2.5
	Neurons	Astrocytes	Microglia	
Co-Con	+	+	-	-
Co-PMB	+	+	-	+
Tri-Con	+	+	+	-
Tri-PMB	+	+	+	+
